# Fruit and Vegetable Pricing by Demographic Factors in the Birmingham, Alabama, Metropolitan Area, 2004-2005

**Published:** 2010-06-15

**Authors:** Jamy D. Ard, Suzanne Perumean-Chaney, Renee Desmond, Bryce Sutton, Tiffany L. Cox, David B. Allison, Frank Franklin, Monica L. Baskin, W. Scott Butsch

**Affiliations:** University of Alabama at Birmingham; University of Alabama at Birmingham, Birmingham, Alabama; University of Alabama at Birmingham, Birmingham, Alabama; University of Alabama at Birmingham, Birmingham, Alabama; University of Alabama at Birmingham, Birmingham, Alabama; University of Alabama at Birmingham, Birmingham, Alabama; University of Alabama at Birmingham, Birmingham, Alabama; University of Alabama at Birmingham, Birmingham, Alabama; MGH Weight Center, Boston, Massachusetts. At the time of this study, Dr Butsch was affiliated with the University of Alabama at Birmingham, Birmingham, Alabama.

## Abstract

**Introduction:**

Fruit and vegetable cost may influence consumption. Because the contextual environment influences food outlet type and availability, we wanted to determine whether neighborhood demographics were associated with prices of fruits and vegetables.

**Methods:**

We surveyed 44 grocery stores in the Birmingham, Alabama, metropolitan area to determine prices of 20 fruits and vegetables. Stores were geocoded and linked to the corresponding Census 2000 block group to obtain data for the independent variables — percentage African American, percentage with at least a high school diploma, and percentage of households below the poverty level. We conducted multiple linear regressions to estimate these predictors for each fruit and vegetable's mean price per serving during 2 seasons (fall/winter 2004, spring/summer 2005).

**Results:**

In the fall, we found no significant relationships between the predictors and prices of any fruits and vegetables in the survey. In the spring, the percentage who had at least a high school diploma was a predictor of price per serving for potatoes (β = 0.001, *P* = .046).

**Conclusion:**

Neighborhood demographics have little consistent influence on fruit and vegetable prices in Birmingham, Alabama, which may be a function of grocery store density, transportation patterns, and shopping patterns. The regional setting of the food environment has implications for food availability, variety, and price.

## Introduction

The presence of different types of food outlets, such as grocery stores, fast-food restaurants, or full-service restaurants, has been associated with demographic characteristics of the populations immediately surrounding the outlets, including race, income, and education (1-5). In addition, the presence or absence of various types of outlets directly affects consumption of food (4,6,7). Consumption of certain food types may also be influenced by marketing and pricing practices of the food outlets. Grocery stores typically have lower prices for most foods than do smaller, independently owned food stores (8,9). Consequently, in addition to the presence of a food outlet, price of food may play a role in consumers' diets. Price may be the mechanism by which increased fruit and vegetable consumption is associated with the presence of grocery stores in a neighborhood (4,5).

Most data supporting this argument have been collected from metropolitan areas, mostly in the northeastern United States, with food outlets such as bodegas, mom-and-pop stores, grocery stores, and warehouse outlets (10-12). We wanted to determine whether the prices of common fruits and vegetables differed across neighborhoods with varying demographic characteristics in Birmingham, Alabama. The Birmingham metropolitan statistical area (MSA) is dominated by large chain grocery stores, and the contextual effects on fruit and vegetable pricing are unclear in this setting. We hypothesized that neighborhood demographic characteristics in the Birmingham MSA, such as percentage African American, percentage with at least a high school diploma, and percentage of households below poverty level, would be associated with variations in fruit and vegetable pricing.

## Methods

The Birmingham MSA has a population of 920,671; 31% of the residents are African American, and 84% have at least a high school diploma (13). We surveyed 44 grocery stores once during each of 2 seasons (fall/winter 2004 and spring/summer 2005) in the Birmingham MSA. The stores were in the same zip codes as the schools participating in the Hi5+ study, a multicomponent school-based intervention to increase fruit and vegetable intake among elementary students in the Birmingham MSA (14).

We selected all grocery stores in the neighborhoods surrounding the 33 elementary schools in the Hi5+ study; we identified stores by zip code from a list obtained from the Alabama Department of Food Safety. We cross-referenced this list with Internet searches and the telephone directory to find missing or new stores. All stores met criteria for retail food stores, as defined by the Food Establishment Sanitation Rules of the Alabama State Board of Health, Bureau of Environmental Sciences (www.jcdh.org/EH/FnL/FnL02.aspx). These criteria are based on the type of food products sold and the level of food handling in the store. We did not include any store that required a membership to shop, such as Costco or Sam's Club.

**Figure 1:**
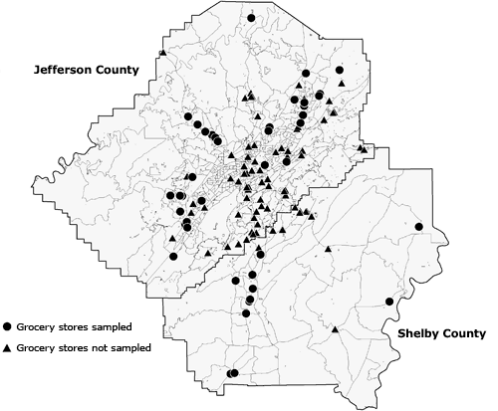
Distribution of 44 sampled and 90 unsampled grocery stores in the Birmingham, Alabama, metropolitan area, 2004-2005.

At the time of data collection, 134 stores were in the Birmingham MSA. The 2-county area of Jefferson and Shelby counties encompasses 1,919 square miles, resulting in 1 store per 14.3 square miles. Of the 134 stores available, we sampled 44 stores (33%) for this study (Figure 1). In our comparison of the neighborhoods surrounding the 90 stores not sampled, the only significant difference in neighborhood demographics was a lower percentage of people with at least a high school diploma in the study sample compared with those not sampled (78% vs 83%, respectively; *t* test statistic *P* = .04).

### Mean price per serving of fruits and vegetables

The top 20 fruits and vegetables eaten away from school by children participating in the Hi5+ study are included in the analysis (Table 1). We chose these foods rather than a composite market basket, such as the US Department of Agriculture’s Thrifty Food Plan (15), for 3 reasons: 1) food preferences, including fruit and vegetable preferences, are influenced by cultural preferences; a generic market basket discounts any cultural preferences for foods (16); 2) since the parents and children reported buying and eating the food locally in the Birmingham MSA, we were sure that they would be accessible in local food stores; and 3) many postulate that the higher cost of fruits and vegetables specifically, rather than other components of a market basket, has contributed to the increase in obesity among children and adults, particularly in lower income groups (17,18).

Trained data collectors used a standardized protocol to collect fruit and vegetable prices. The nondiscounted price was collected as the primary outcome for each fruit and vegetable. Fresh fruits and vegetables excluded from data collection included those labeled "organic," those that were prewashed or prepackaged, and mixed food items that were bundled together (for example, a bag containing both apples and oranges). We excluded special-preparation items (no salt added [because sodium is typically added in processing canned vegetables], sauce added) and vegetable or fruit mixtures that were not specifically identified by the Hi5+ study. We included canned and frozen fruits and vegetables in this study because we had no information to differentiate the type of food the child reported eating. We collected all brand names for a given canned or frozen item, using a standard package size as the reference (typically 14.5-oz cans and 1-lb bags). We calculated cost per serving by dividing the total cost for 1 item by the number of servings. Servings for each item were based on the weight for each fruit and vegetable and were obtained from the US Department of Agriculture Economic Research Service (www.ers.usda.gov/Data/Fruitvegetablecosts/).

### Geocoding and socioeconomic status variables

The 44 sampled grocery stores were geocoded to the Census 2000 TIGER/line data by using ArcGIS version 9.1 (ESRI, Redlands, California). Each store's location was verified by using a combination of Google Earth and MapQuest. The grocery stores were then mapped to the Census 2000 at the block group level. The block group level is 1 level above the census blocks and contains 250 to 550 housing units (mean, 400) and approximately 600 to 3,000 people. Each census tract contains 9 block groups. The block group was selected because it was the smallest census level available for the independent variables of interest. The 3 socioeconomic status (SES) variables chosen as the predictor variables were obtained from the Census 2000 Summary File 3 (SF3). The variables included the percentage of the population that was African American, the percentage of adults aged 25 years or older with a high school diploma or equivalent, and the percentage of households with income in 1999 below the poverty level. All maps were produced with ArcGIS.

### Data analysis

We used independent *t* tests to examine the difference between the sampled and nonsampled stores with respect to the SES variables. We used analysis of variance to determine differences in the unadjusted mean prices between tertiles of each demographic factor and used the Tukey post hoc test when there was a significant overall *F* statistic (*P* < .05). To assess the primary study outcome, multiple linear regression estimated the effect of the predictor SES variables on the price per serving of each fruit and vegetable during the fall/winter and spring/summer seasons. Diagnostics for all the multiple regression models were assessed with residual plots and tests for normality. The variance inflation factor and tolerance were used to test for multicollinearity. All analyses were conducted with SPSS version 13 (SPSS, Inc, Chicago, Illinois).

## Results

**Figure 2:**
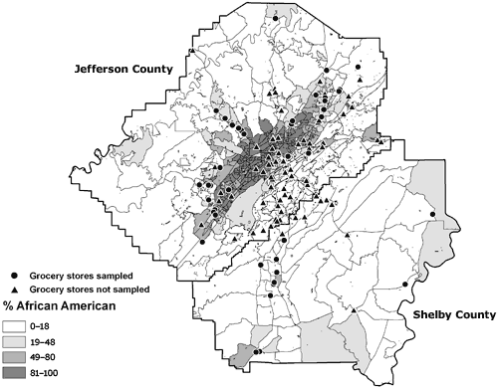
Locations of 44 sampled and 90 unsampled grocery stores in the Birmingham, Alabama, metropolitan area and percentage of African American residents, by census block group, 2004-2005.

The stores were located throughout Jefferson and Shelby counties. The median (interquartile range [IQR]) percentage of residents in the neighborhoods around the stores that were African American was 29%, and 7 stores were in majority African American neighborhoods. The median percentage of people with at least a high school diploma was 80%, and the median percentage of households below the poverty level was 12% (Table 2). Figures 2 through 4 depict the location of the sampled stores in relation to the SES variables — percentage African American, percentage with a high school diploma, and percentage of households below the poverty level, respectively.

**Figure 3:**
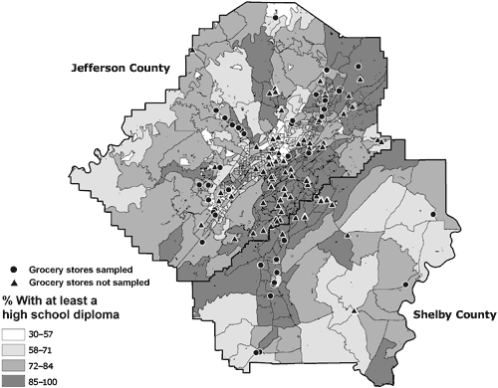
Locations of 44 sampled and 90 unsampled grocery stores in the Birmingham, Alabama, metropolitan area and percentage of residents who have at least a high school diploma, by census block group, 2004-2005.

**Figure 4:**
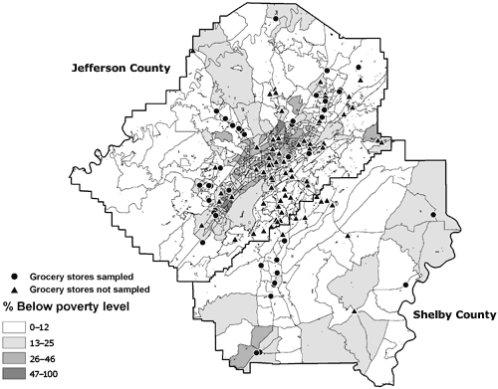
Locations of 44 sampled and 90 unsampled grocery stores in the Birmingham, Alabama, metropolitan area and percentage of residents who live below the poverty level, by census block group, 2004-2005.

Strawberries and grapes were most expensive during the fall, and carrots and potatoes were least expensive (Table 1). In the spring, the price of strawberries decreased an average of $0.42, yet they were still among the most expensive items. Oranges and grapes were also in the higher price range during the spring. The price of potatoes declined in the spring, making them the cheapest of the 20 fruits and vegetables.

For the fall, grapes were the only fruit or vegetable that was significantly different when comparing the highest ($0.60) to lowest ($0.47) tertile for percentage with at least a high school diploma (*P* < .05). In the spring, the price per serving for potatoes was significantly different between the highest and lowest tertiles for percentage with at least a high school diploma ($0.13 vs $0.09, *P* = .002) and percentage below the poverty level ($0.09 vs $0.13, *P* = .004). Strawberry prices were also significantly different in the spring when comparing the middle and top tertiles for percentage below the poverty level: the middle tertile had the higher cost per serving ($0.16, *P* = .048).

**Figure 5:**
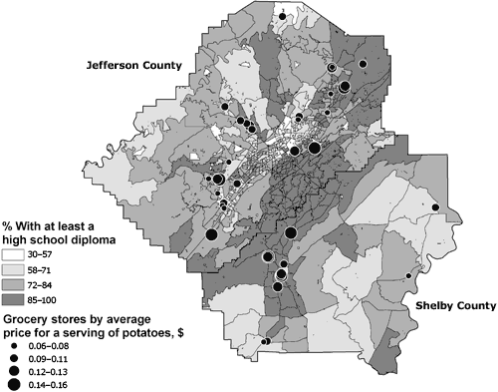
Variation in the price of potatoes at grocery stores in the Birmingham, Alabama, metropolitan area and percentage of residents who have at least a high school diploma, by census block group, 2004-2005.

In general, only 1 of the 20 fruits and vegetables had any relationship with neighborhood demographics, and that relationship was valid only during the spring. The average price of potatoes during the spring months was significantly predicted by the percentage of people who had at least a high school diploma (Table 3). That is, as the number of people with a high school diploma increased by 1 percentage point, the price per serving of potatoes increased by an average of $0.001, controlling for the other SES variables (Figure 5).

## Discussion

Despite demographic variation, we observed virtually no effects on fruit and vegetable prices of the percentage of the population that was African American or of education or poverty level across the 44 stores we surveyed in the Birmingham MSA. Our findings suggest that relationships between fruit and vegetable pricing and neighborhood demographics are limited in the Birmingham area.

The characteristics of this study may influence the findings in a number of ways. Factors such as grocery store density and transportation patterns are unique to a given area and could affect fruit and vegetable prices. With the dominance of large chain grocery stores in the Birmingham MSA, not only are prices suppressed and retailer competition increased, but the ability of a smaller grocer to maintain a viable business may be limited because of pressure to lower prices. As a result of their proximity to each other, large stores provide the consumer with multiple shopping options. Other factors to consider include the transportation patterns of adults in the Birmingham area. According to the 2000 census, 94% of households in the area have at least 1 car, and 40% of households have 2 cars. Driving allows consumers to travel farther from home, increasing the available food outlet options.

One key point of this study is that the regional setting of the food environment seems to affect food availability, variety, and pricing. Across different types of population centers (for example, rural compared with urban), previous studies have shown differences in the types and density of food outlets available. One study in the Lower Mississippi Delta region found a low density of large grocery stores and supermarkets (8). Of 36 counties studied, there was only 1 store per 1.6 square miles. In the urban Birmingham area, there is 1 store per 14.3 square miles. Given the low accessibility of larger stores in rural areas, smaller food outlets play a larger role in the food supply. These smaller stores typically charge 3% to 10% more for food (8). Even when people in rural areas access larger stores, their travel costs may be substantially higher than those of people who live in urban areas.

Regional differences are also apparent when assessing the food environment, even in population centers designated as urban. For example, 1 study reported a total of 324 food stores in East Harlem and the Upper East Side of New York City (12). Despite the high concentration of stores in this densely populated area, 249 of the stores (77%) had only 1 cash register, indicating a smaller food store with less variety. However, in our local assessment, almost all of the stores had more than 4 cash registers, and there were no major differences in the variety of items available within the stores.

In our limited regional setting, the lack of variation in food price has at least 2 implications. First, this evidence does not support the notion that obesity is related to high prices of fruit and vegetables. Despite the high availability and lack of price variation for fruits and vegetables, the prevalence of obesity was approximately twice as high in African American parents of children from the Hi5+ study as in non-African Americans (16). The second implication of this study is that it may highlight an opportunity to increase fruit and vegetable intake by increasing knowledge about the availability of healthy alternatives that are reasonably priced. To take advantage of a food environment that offers a variety of fruits and vegetables, additional effort will need to be directed toward helping people make healthy choices in an economical fashion.

This study has several limitations. Although we did not see differences across neighborhoods in prices of fruits and vegetables in the surveyed stores, additional factors may increase food costs for those who have lower income, are African American, or have less education. One study showed that impoverished neighborhoods that were predominately African American were approximately 1.1 miles farther from the nearest grocery store than were white neighborhoods (11). Travel distance was not assessed in our study and may be a moderating factor that increases the cost of food. Differences in food quality may also affect cost in a way that is not reflected in retail price. If the quality of an item is better at 1 store than at another, even if the price is the same, the perceived value of the higher-quality item will be higher. Quality, a highly subjective measure, was not assessed in this study and may vary by the demographic variables of interest. Store-level factors and potential spatial inequalities should be considered as well. We presumed that the density of stores, services offered, and size of the stores are relatively constant across the sites because they had to meet the criteria for a retail food store. Unaccounted differences may contribute to variations in price of or access to fruits and vegetables. Spatial inequalities in metropolitan areas such as Birmingham can also lead to variations that may not be captured in our study design. Relevant types of spatial inequalities include the tendency for large chain grocery stores to locate in more affluent areas, along with more availability of green spaces and full-service restaurants that may actively support a healthy lifestyle and healthful food choices.

In conclusion, our findings suggest that in the Birmingham MSA, where there is a high density of grocery stores that sell fruits and vegetables, neighborhood demographics are not associated with variations in fruit and vegetable prices. Despite the high availability of fruits and vegetables and the lack of price variations for them, intake remains lower than recommended. Interventions in the Birmingham MSA aimed at educating people about the availability and economical choices of fruits and vegetables could be effective at reducing obesity. For example, point-of-purchase programs that incorporate education on food labeling as well as shelving-level promotion of fruits and vegetables have shown promise in increasing consumption of fruits and vegetables, particularly among minority populations (19,20). Additionally, educating consumers on the value of fruits and vegetables could be done through in-store cooking demonstrations and signs that show a product comparison of various healthy alternatives at multiple price points. To more fully understand the dynamics of decision-making processes involved with buying and eating fruits and vegetables, research is needed to address perceived obstacles, including availability, preparation time, and product uses, in conjunction with the price of fruits and vegetables.

## Figures and Tables

**Table 1:** Average Price per Serving by Season and Type of Fruit or Vegetable Among Grocery Stores in Birmingham, Alabama, 2004-2005

Item	Mean (Standard Deviation) Price, $^a^		

	Fall	Spring	Mean Annual Price
Apple juice	0.18 (0.03)	0.18 (0.03)	0.18 (0.03)
Apples	0.16 (0.03)	0.16 (0.03)	0.16 (0.03)
Applesauce (fall n = 39)	0.24 (0.03)	0.25 (0.03)	0.24 (0.03)
Bananas (fall n = 43, spring n = 38)	0.17 (0.02)	0.18 (0.02)	0.18 (0.02)
Broccoli	0.18 (0.04)	0.17 (0.03)	0.18 (0.04)
Carrots	0.13 (0.02)	0.15 (0.05)	0.14 (0.04)
Corn	0.35 (0.05)	0.33 (0.06)	0.34 (0.06)
Fruit cocktail (fall n = 43)	0.31 (0.03)	0.31 (0.03)	0.31 (0.03)
Grape juice	0.28 (0.03)	0.27 (0.03)	0.28 (0.03)
Grapes (fall n = 41, spring n = 38)	0.54 (0.14)	0.46 (0.12)	0.50 (0.13)
Green beans	0.24 (0.05)	0.23 (0.05)	0.23 (0.05)
Green peas	0.37 (0.08)	0.35 (0.07)	0.36 (0.08)
Lettuce	0.23 (0.13)	0.26 (0.11)	0.24 (0.12)
Orange juice	0.24 (0.04)	0.23 (0.03)	0.24 (0.03)
Oranges (fall n = 43)	0.52 (0.20)	0.57 (0.51)	0.54 (0.38)
Pears (fall n = 42)	0.28 (0.03)	0.28 (0.03)	0.28 (0.03)
Pineapple (fall n = 43, spring n = 38)	0.26 (0.03)	0.26 (0.04)	0.26 (0.04)
Potatoes, white	0.14 (0.11)	0.11 (0.03)	0.13 (0.08)
Strawberries (fall n = 31, spring n = 33)	0.89 (0.16)	0.47 (0.16)	0.67 (0.26)
Tomatoes (fall n = 43)	0.51 (0.16)	0.43 (0.20)	0.47 (0.18)

a Except where noted otherwise, the number of stores surveyed was 44 in the fall and 39 in the spring.

**Table 2:** Neighborhood Characteristics by Census Block Group, Birmingham, Alabama, 2004-2005

Block Group^a^	No. of Stores	% (Standard Deviation)		

		African American	Have at Least High School Diploma	Households Below Poverty Level
1	18	16 (21)	83 (6)	9 (5)
2	6	11 (10)	80 (10)	9 (8)
3	6	27 (18)	73 (14)	13 (13)
4	4	25 (16)	81 (6)	12 (5)
5	5	48 (42)	59 (17)	23 (14)
6	2	40 (9)	76 (17)	21 (20)
9	3	34 (35)	84 (7)	6 (3)

a No stores were sampled in block groups 7 and 8.

**Table 3:** Multiple Regression for the Average Price of Potatoes in the Spring, Birmingham, Alabama, 2005^a^

**Predictor**	β	Standard Error (β)	*b*	*t*	*P* Value
% African American	0.000	0.000	−0.014	−0.063	.95
% With at least high school diploma	0.001	0.001	0.527	2.068	.046
% Households below poverty level	0.000	0.001	0.032	0.099	.92

a *F*_(35,3)_ = 4.073; *P* = .014; *R* ^2^ = 0.259; adjusted *R* ^2^ = 0.195; standard error of the estimate, 0.026.
